# Enhancement or suppression: the impact of social media “fertility fear emotion” on college students’ fertility intentions

**DOI:** 10.3389/fpsyg.2026.1930938

**Published:** 2026-09-01

**Authors:** Zhi-Hong Xu, Chang-hua Liu, Yong-Xian Liu, Gang Cao

**Affiliations:** 1 School of Marxism, Communication University of Zhejiang, Hangzhou, China; 2 School of International Education, Communication University of ZheJiang, Hangzhou, China; 3 College of Teacher Education, Yangtze Normal University, Chongqing, China; 4 Hangzhou Chengxi Middle School, Hangzhou, China

**Keywords:** college students, enhancement or suppression, fertility fear emotion, fertility intentions, social media

## Abstract

**Objective:**

The fertility fear emotion spread via social media has gradually become a prevalent social phenomenon. College students constitute the main fertility force in the future, and improving their fertility intentions has become an urgent issue to be addressed. This study aims to explore the impact of social media-induced fertility fear emotion on college students’ fertility intentions and reveal its underlying mechanism.

**Methods:**

An online survey was conducted among 1,036 participants using questionnaires including the Social Media Fertility Fear Scale, Fertility Intention Scale, Fertility Attitude Scale, Subjective Norms Scale, and Perceived Behavioral Control Scale. A total of 979 valid responses were collected. Descriptive statistical analysis, correlation analysis, and the Bootstrap method were adopted for data analysis.

**Results:**

(1) Fertility fear emotion was significantly negatively correlated with fertility attitudes, subjective norms, perceived behavioral control and fertility intentions. Fertility intentions were significantly positively correlated with subjective norms, perceived behavioral control and fertility attitudes. (2) Fertility attitudes, subjective norms and perceived behavioral control exerted a full mediating effect between social media fertility fear emotion and fertility intentions. There existed a chain mediation effect among the three variables: perceived behavioral control affected subjective norms, which further influenced fertility attitudes.

**Conclusion:**

Within the framework of the Theory of Planned Behavior, the relationship between social media fertility fear emotion and college students’ fertility intentions is mediated by fertility attitudes, subjective norms and perceived behavioral control. College students should treat fertility-related information on social media rationally.

## 1 Introduction

A global trend of low fertility rates persists, posing significant demographic challenges. In response, China has successively introduced policies aimed at boosting birth rates and enhancing population dynamics. The “selective two-child” policy was implemented in 2014, followed by the “universal two-child” policy in 2016, and most recently, the “three-child policy” in 2021. However, despite these policy interventions, birth rates have continued to decline steadily. Data from the National Bureau of Statistics reveals that China’s birth population has experienced a consecutive decrease over the past 7 years, starting from 2017, and culminating in negative population growth in both 2022 and 2023. Although a temporary resurgence was observed in 2024, with an increase of 520,000 newborns, largely attributed to cultural preferences for “dragon year” births and policy incentives, the underlying challenges of an aging population and persistently low fertility rates remain.

Macroscopically, fertility is shaped by policy, economy, and culture. Microscopically, individual fertility intentions, subjective attitudes toward childbearing, serve as key predictors of fertility behavior and population trends (Kuhnt and Trappe, 2016). The fertility intentions of young adults significantly influence national fertility rates. Research indicates that economic pressures, childcare burdens, and educational expenses contribute to a “low-fertility culture” among Chinese youth (Wu, 2020).

Furthermore, social media has reshaped lifestyles and marital values. Although governments and mainstream media promote pronatalist policies, the decline in fertility intentions, coupled with the rising phenomenon of “Fertility Fear Emotion” on social media, contradicts these efforts. “Fertility Fear” refers to the emotional experience in which women develop worries, fears, or even a desire to evade when contemplating the prospect of childbearing in the future. Social media amplifies collective anxieties regarding childbirth, which may influence the fertility intentions of youth—a factor underexplored in existing literature. This study integrates social psychology and fertility theories to quantify the relationship between social media’s “Fertility Fear Emotion” and college students’ fertility intentions, providing insights to address the decline in fertility rates in China.

## 2 Theoretical framework and hypotheses

### 2.1 Social media’s impact on fertility intentions

In the era of social media, the affordances of new technologies have fundamentally reshaped the media communication ecosystem, emerging as a transformative force that reconstructs individual perceptions and behaviors. Social media processes and reframes fertility-related information, particularly information about reproductive risks, amplifying societal effects through both mass and interpersonal communication channels. However, academic conclusions regarding the impact of social media on fertility intentions remain inconsistent. Some researchers argue that the echo chambers created by social media weaken individual childbearing intentions, with cognitive biases and public opinion crises triggered by fertility risk information exacerbating reproductive anxiety (Liu and Song, 2022). For instance, Qiu et al. (2022) found that internet users exhibit approximately 10.5% lower fertility intentions compared to non-users. Guldi and Herbst (2017) analyzed adolescent fertility rates from 1999 to 2007, concluding that increased internet usage contributed to a 7% reduction in birth rates. Liu et al. (2021) also discovered that frequent social media users tend to have lower fertility intentions and weaker adherence to traditional gender roles. Conversely, Billari et al. (2009) found that social media enhanced fertility levels among German women by helping individuals balance family and work commitments. Scholars such as Francesco et al. (2019) proposed a dual-effect theory: while social media may promote fertility by facilitating work-life balance, its capacity to rapidly disseminate negative fertility narratives could simultaneously suppress childbearing intentions.

### 2.2 Social media’s fertility fear emotion

Social media serves as a vital tool in shaping societal attitudes by influencing individuals’ fertility perceptions through the interactive dynamics of disseminating childbearing-related information (Liu and Song, 2022). Witteman et al. (2016) discovered that exposure to one-sided social media commentaries with biased perspectives significantly impacts people’s views on family reproduction. Scholars analyzing informational elements in online texts observed that “fertility threats” have become predominant in social media narratives (Yang and Wang, 2023). When individuals evaluate the costs and benefits of childbearing, online information tends to exacerbate perceived negative effects, fostering a climate of “childbearing fear” (Guldi and Herbst, 2017). Stoll et al. (2014) revealed that young women express greater concern than young men regarding bodily changes following post-pregnancy and childbirth, with students who learn about reproduction through media reporting the highest levels of fear. This “childbearing apprehension” functions like an “invisible hand” continuously triggering fertility-related sensitivities among youth. The repetitive reinforcement and diffusion of such fears expand their influence, creating a vortex of reproductive anxiety. This pervasive fear may deminish individual fertility intentions, ultimately exerting negative consequences on birth rates.

### 2.3 Mediating role of the Theory of Planned Behavior (TPB)

The Theory of Planned Behavior (TPB), a framework derived from social psychology that seeks to explain and predict human behavior, posits that attitude toward behavior (ATB), subjective norms (SN), and perceived behavioral control (PBC) are psychological factors influencing behavioral intentions and actions. This theory has been widely applied in various fields, including healthcare, environmental science, and education (Duan and Jiang, 2008). Additionally, scholars both domestically and internationally have adopted TPB to study fertility intentions and behaviors, supported by both quantitative and qualitative evidence. However, there remains some controversy regarding its applicability. In a cross-national study on fertility intentions, Ajzen and Klobas (2013) identified ATB, SN, and PBC as critical determinants of childbearing intentions. Similarly, research by Mencarini et al. (2015) in Italy and Dommermuth et al. (2017) in Norway confirmed that attitudes, subjective norms, and perceived behavioral control are decisive factors: the more positive the fertility attitudes, the stronger the perceived normative pressures and behavioral control, the higher fertility intentions among individuals. However, the findings of Zhou et al. (2021) diverged, indicating that ATB significantly influences fertility intentions, SN has no significant effect, and PBC exerts a highly significant positive influence. Matera et al. (2023) argued that some variables have no correlation with fertility intentions, and the correlation strength between variables varies substantially. Although the Theory of Planned Behavior remains a robust framework for analyzing fertility intentions, its application in examining how social media environments shape individual childbearing intentions is still underexplored.

### 2.4 Research framework

Drawing upon the literature reviewed above, this study employs a socio-psychological perspective to investigate the impact of “Fertility Fear Emotion” on social media on fertility intentions. Based on this foundation, the study proposes the following four hypotheses:

*Hypothesis 1*: Social media-induced “Fertility Fear Emotion” has a negative influence on the fertility intentions of college students.

*Hypothesis 2*: The three factors of the Theory of Planned Behavior (TPB), namely attitude toward the behavior, subjective norms, and perceived behavioral control, collectively mediate the relationship between social media-induced “Fertility Fear Emotion” and fertility intentions.

*Hypothesis 3*: Subjective norms play a mediating role between the fear of having children on social media and fertility intentions.

*Hypothesis 4*: Perceived behavioral control mediates the relationship between the fear of having children on social media and fertility intentions.

**Figure 1:**
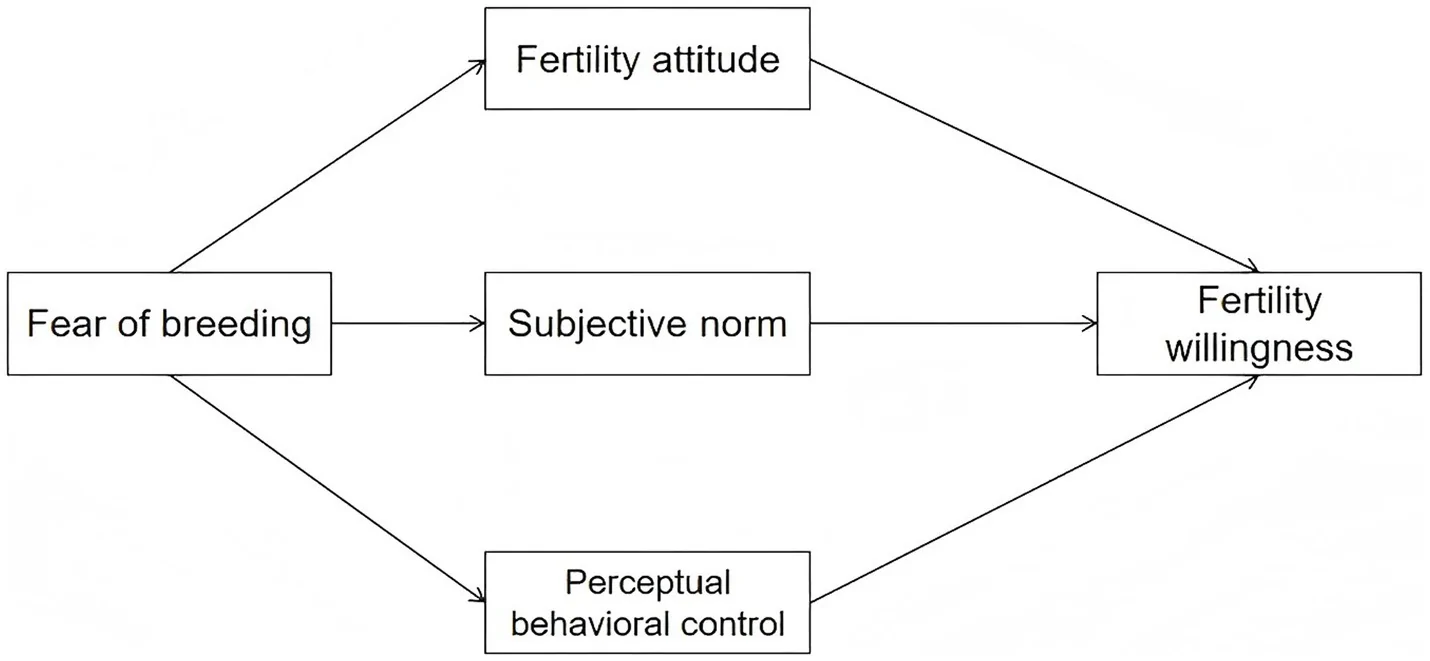
Theoretical hypothesis model.

By elucidating the influence mechanism of the fear of parenthood portrayed on social media on fertility intentions, this study establishes a comprehensive mediating model (as shown in Figure 1).

## 3 Research methods

### 3.1 Questionnaire design

The questionnaire is divided into two main sections: the first part assesses the demographic characteristics of college students, covering aspects such as gender, academic year, whether they are the only child, average daily internet usage time, etc. The second part focuses on the measurement of five latent variables: fear of childbearing emotion, fertility attitudes, subjective norms, perceived behavior control, fertility intentions. All variables were measured using a five-point Likert scale, with scores ranging from1 (indicating very inconsistent) to 5 (indicating very consistent). To ensure the reliability and validity of the questionnaire, a pre-tested was conducted on a small scale before formal release. Prior to formal distribution, all questionnaires underwent preliminary testing on a small scale and were subsequently refined to create the final versions for widespread dissemination.

### 3.2 Research objects

This study involved college students as participants, utilizing a convenience sampling method. Data collection was conducted through the online survey platform of Wenjuanxing. The samples primarily consisted of individuals from Zhejiang, Hunan, Chongqing, Shandong and other regions. A total of 1,036 responses were received, of which 979 were deemed valid after removing responses with less than 50 s of completion time and those containing identical answers across all questions, which accounted for more than 10 times the number of analyzed items. The final sample size was appropriate, with a data efficiency rate of 94.5%. Descriptive statistical analysis was performed on the 979 valid responses to examine the sample’s characteristics, as detailed in Table 1.

**Table 1:** Survey sample population characteristics.

Variable	Option	Frequency	Percentage (%)
Gender	Male	437	44.64
	Female	542	55.36
Family background	Rural family	386	39.43
	Township family	171	17.47
	Urban family	422	43.11
Only child	Yes	341	34.83
	No	638	65.17
Grade	Year 1	305	31.15
	Year 2	123	12.56
	Year 3	507	51.79
	Year 4	44	4.49
Parental education	High school or below	711	72.63
	Bachelor’s degree	260	26.56
	Master’s/doctoral degree	8	0.82
Social media usage duration	0–3 h	231	23.6
	3–6 h	399	40.76
	Over 6 h	349	35.65

### 3.3 Research tools

#### 3.3.1 Fertility fear emotion questionnaire

Drawing on the research of Wan and Zhou (2023), this study designed a questionnaire to assess the “Fertility Fear Emotion,” focusing on the impact of social media content related to birth pain, education cost, and improper parenting. The questionnaire includes four items, such as “social media descriptions of the cost of childbearing and the responsible parenting make me fear having children.” The internal consistency coefficient is 0.920.

#### 3.3.2 Fertility intention questionnaire

This questionnaire measures the external pressures individuals perceive when considering fertility issues. When making reproductive decisions, individuals may consider the wishes of their close associates or internalize fertility concepts from social media to form their own reproductive desires (Li and Wang, 2021). The questionnaire includes three items, such as “My parents want me to have children.” Higher scores indicate a greater willingness to have children. The internal consistency coefficient for this scale is 0.760.

#### 3.3.3 Three-dimensional questionnaire of planned behavior theory

According to the planned behavior assessment scale (Ajzen, 1991) compiled by Ajzen, the author of the planned behavior theory, the following questionnaire was compiled by referring to the indicators selected in existing literatures and combining with interviews with college students:

*Fertility attitude questionnaire*: Incorporating indicators from existing literature and interviews with college students, this questionnaire assesses an individual’s value judgment and consequence assessment of reproductive behavior. A more positive fertility attitude is associated with stronger fertility intentions. The questionnaire includes six items, such as “I think having children will bring me many restrictions.” Higher scores indicate a more negative evaluation of fertility. The internal consistency coefficient is 0.907.

*Subjective normative questionnaire*: This questionnaire measures the external pressures individuals perceive when considering fertility issues. When making reproductive decisions, individuals may consider the wishes of their close associates or internalize fertility concepts from social media to form their own reproductive desires (Li and Wang, 2021). The questionnaire includes three items, such as “My parents want me to have children.” Higher scores indicate a greater willingness to have children. The internal consistency coefficient for this scale is 0.760.

*Perceived Behavior control questionnaire*: This questionnaire assesses the perceived difficulty of reproductive behavior, primarily derived from conditions that promote or hinder such behavior (such as economic conditions, resources, abilities). The questionnaire was designed from the perspectives of economic conditions, self-efficacy, and marital feelings, with a total of four items, such as “If the partner can bear the responsibility of raising children, they can consider having children.” Higher scores indicate a greater willingness to have children. The internal consistency coefficient for this scale is 0.901.

### 3.4 Data processing

To ensure the validity and scientific rigor of the data analysis, SPSS 25.0 was employed to conduct descriptive statistics, correlation analysis and reliability analysis. Additionally, AMOS24.0 was utilized to construct a structural equation model, which allowed for the estimation of the model fit and the examination of the relationships among the key variables.

## 4 Data analysis results

### 4.1 Common method deviation detection

Given that all data were collected via self-report questionnaires distributed to college students, the issue of common method bias is inevitable. Following the recommendations of Podsakoff et al. (Podsakoff et al., 2003), we adopted the unmeasured latent method factor approach to test for common method bias. The results showed that adding one method factor to the five-factor model to form a six-factor model yielded no substantial improvement in model fit indices (ΔCFI = 0.01, ΔTLI = 0.01, ΔRMSEA = 0.01, ΔSRMR = 0.03). In addition, the results of confirmatory factor analysis (CFA) revealed poor fit for the single-factor model (*χ*^2^ = 5283.76, *p* < 0.001; CFI = 0.67; TLI = 0.64; RMSEA = 0.17; SRMR = 0.10). Accordingly, it can be concluded that common method bias is not a serious concern in this study.

### 4.2 Correlation analysis

The correlation analysis results revealed several significant relationships among the variables (as shown in Table 2). Specifically, there was a significant negative correlation between fertility fear emotion and fertility intention (*r* = −0.469, *p* < 0.001), which proved hypothesis 1. Additionally, there was a significant negative correlation between fertility fear emotion and fertility attitude (*r* = −0.723), subjective norms (*r* = −0.451, *p* < 0.001) and perceived behavioral control (*r* = −0.411, *p* < 0.001). This shows that the higher the fertility fear emotion of college students, the lower their attitude towards childbearing, subjective norms and perceived behavior control; There were significant positive correlations between subjective norms and reproductive intention (*r* = 0.708, *p* < 0.001), perceived norms of behavior and reproductive intention (*r* = 0.709, *p* < 0.001), and reproductive attitude and reproductive intention (*r* = 0.578, *p* < 0.001). These results suggest that stronger subjective norms, higher perceived behavioral control, and more positive fertility attitudes are all associated with higher fertility intentions.

**Table 2:** Discriminant validity of variables and common method bias test.

Model	*χ* ^2^	*df*	*χ*^2^/*df*	CFI	TLI	RMSEA	SRMR
Single-factor model	5283.76	189	27.96	0.67	0.64	0.17	0.10
Two-factor model	3731.37	188	19.85	0.77	0.75	0.14	0.09
Three-factor Model	1851.64	186	9.96	0.89	0.88	0.10	0.06
Four-factor model	1569.65	183	8.58	0.91	0.90	0.09	0.07
Five-factor model	959.41	179	5.36	0.95	0.94	0.07	0.06
Five-factor model + latent method factor	727.22	159	4.57	0.96	0.95	0.06	0.03

ky = Fertility Fear Emotion; td = Fertility Attitudes; zg = Subjective Norms; kz = Perceived Behavioral Control; yy = Fertility Intentions.

Single-factor model: ky + td + zg + kz + yy Two-factor model: ky; td + zg + kz + yy Three-factor model: ky; td; zg + kz + yy Four-factor model: ky; td; zg; kz + yy Five-factor model (construct measurement model): ky; td; zg; kz; yy.

### 4.3 Model test and path analysis

Based on the theoretical framework of planned behavior and the corresponding variable design, it is evident that the influence of “childfear” emotion on college students’ reproductive intention involves a complex, multi-effect relationship among multiple potential variables. This relationship was analyzed using the structural equation model (SEM), constructed with AMOS24.0. The latent variables in the model include fertility fear emotion, fertility attitude, subjective norms, perceived behavior control and fertility intention, all of which are measured by 21 observational variables. The causal paths between observed variables and latent variables form the measurement relationship of the model, while the correlation among fertility attitudes, subjective norms and perceived behavioral control constitutes the covariant relationships.

Given the characteristics of the data, the SEM was constructed using AMOS software, with model estimation conducted via the maximum likelihood method. This study hypothesized that fertility attitudes, subjective norms and perceived behavior control affect college students’ reproductive intentions and mediates the impact of fertility fear emotion on the reproductive intention. At the same time, the fitting parameters of the structural equation model were estimated, yielding the following fit indexes: *χ*^2^/*df* = 5.321, GFI = 0.915, AGFI = 0.891, CFI = 0.950, RMSEA = 0.066. According to the basic standards for model fit, the RMSEA value should be less than 0.1, while GFI and CFI should be greater than 0.9. Based on these criteria, the overall fit of the model is deemed satisfactory.

The analysis began with significance tests for each path coefficient in the model, followed by tests for mediating effects. The results are as follows.

**Figure 2:**
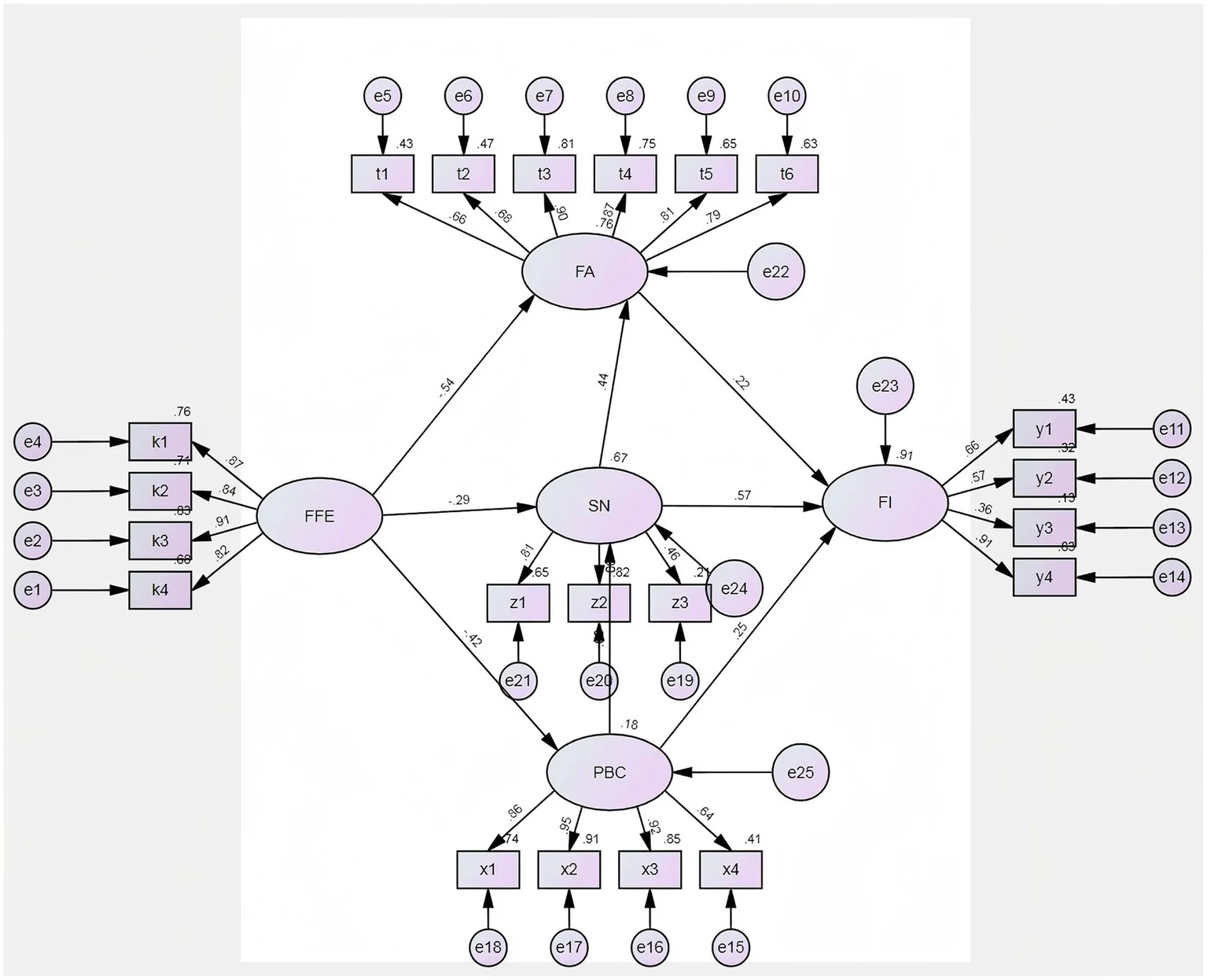
Structural equation model of influencing factors of fertility intention. FFE, fertility fear emotion; FA, fertility attitudes; SN, subjective norms; PBC, perceived behavioral control; FI, fertility intentions.

As depicted in Figure 2, “fear of parenthood” exerts a significant negative influence on fertility attitude (*β* = −0.54, *p* < 0.01). The standardized path coefficient indicates that “fear of parenthood” has the strongest impact on college students’ fertility attitude among the examined factors, with a standardized coefficient of −0.54. Specifically, a one percentage point increase in fertility fear emotion directly reduces childbearing attitudes by 0.54 percentage points. Additionally, fertility fear emotion has a significant negative effect on perceived behavioral control (*β* = −0.42, *p* < 0.01), with a standardized path coefficient of −0.42. This suggests that a one percentage point increase in fertility fear emotion would directly diminish fertility attitude by 0.42 percentage points. The fertility fear emotion had a significant negative impact on the subjective norm (*β* = −0.29, *p* < 0.01), albeit with the lowest standardized path coefficient of −0.29, indicating that an increase of one percentage point in the fertility fear emotion would directly reduce the fertility attitude by 0.29 percentage points.

Among the three cognitive dimensions influencing fertility intention, subjective norm has the highest standardized path coefficient of 0.57. This implies that one percentage point increase in subjective norm directly boosts fertility intention by 0.57 percentage points, highlighting that subjective norm is the primary driver of fertility intention. The standardized path coefficient for perceptual behavior control is 0.25, while that for fertility attitude is the lowest (0.22). These findings suggest that college students place greater emphasis on fertility information from social media and fertility opinions of important individuals in their social circles.

### 4.4 Significance test of mediating effect

To assess the significance of the mediating effects in the model, this study analyzed both the indirect effect and direct effect of the mediating mechanisms. The AMOS Bootstrap method was employed, with 1,000 bootstrap samples randomly and repeatedly drawn to estimate the confidence interval of the mediation effects. The results of mediation analysis are presented in Table 3.

**Table 3:** Descriptive statistics and correlative analysis results.

variable	Fertility fear emotion	Subjective norms	Perceived behavioral control	Fertility attitudes	Fertility intentions
“Fertility fear” emotional	1				
Subjective norms	−0.451^**^	1			
Perceived behavioral control	−0.411^**^	0.728^**^	1		
Fertility attitudes	−0.723^**^	0.599^**^	0.533^**^	1	
Fertility intentions	−0.469^**^	0.708^**^	0.709^**^	0.578^**^	1
Mean (M)	11.93	9.99	14.14	17.28	12.46
Standard deviation (SD)	4.89	2.76	3.84	5.98	3.52

In correlation analysis and SPSS output: ** = *p* < 0.01, significant correlation at the 0.01 level (two‑tailed test).

Specifically, the mediating effect of fertility fear emotion affecting fertility intention through fertility attitude was found to be −0.078, with a 95% confidence interval (CI) of [−0.113, −0.047]. Since this interval does not include zero, Hypothesis H2 is supported as valid. Additionally, the mediating effect of the fertility fear emotion on fertility intention through subjective norms is −0.106, with a 95% CI is [−0.152, −0.072]. Again, the absence of zero in this interval confirms the validity of Hypothesis H3. Lastly, the mediating effect value of fertility fear emotion on fertility intention through perceived behavior control was −0.068, and 95% CI of [−0.101, −0.043]. This further supports Hypothesis H4 as valid, given that the interval excludes zero (Table 4).

**Table 4:** Effects of mediation model.

Pathand effect path and effect	Estimate	Bootstrap (1,000 times) 95% CI			
		Deviation correction		Percentile	
		Lower limit	Upper limit	Lower limit	Upper limit
“Fertility fear” emotional → perceived behavioral control	−0.420	−0.484	−0.354	−0.482	−0.351
“Fertility fear” emotional → subjective norms	−0.286	−0.352	−0.225	−0.351	−0.222
“Fertility fear” emotional → fertility attitudes	−0.545	−0.605	−0.480	−0.604	−0.477
Fertility attitudes → fertility intentions	0.439	0.353	0.512	0.361	0.518
Subjective norms → fertility intentions	0.220	0.135	0.303	0.138	0.304
Perceived behavioral control → fertility intentions	0.573	0.437	0.707	0.442	0.712
“Fertility fear” emotional → fertility attitudes → fertility intentions (indirect effect)	−0.078	−0.113	−0.047	−0.112	−0.047
“Fertility fear” emotional → subjective norms → fertility intentions (indirect effect)	−0.106	−0.152	−0.072	−0.149	−0.071
“Fertility fear” emotional → perceived behavioral control → fertility intentions (indirect effect)	−0.068	−0.101	−0.043	−0.097	−0.040
Perceived behavioral control → subjective norms → fertility attitudes	0.298	0.237	0.361	0.238	0.364
Total effect: “fertility fear” emotional → fertility intentions	−0.602	−0.654	−0.541	−0.654	−0.542

Moreover, this study examined the interactions among fertility attitude, subjective norm and perceived behavior control in mediating the relationship between fertility fear emotion and fertility intention. The results revealed a chain mediation effect among these three variables: perceived behavioral control influences subjective norm, which in turn affects fertility attitude. The effect size of this chain mediation was 0.298, with the 95% CI was [0.237, 0.361]. Overall, the total mediating effect of the entire model is −0.602, with a 95% CI of [−0.654, −0.541]. Since this interval does not include zero, indicating that the three dimensions of planned behavior theory play a complete mediating role in the relationship between fertility fear emotion and fertility intention on social media.

## 5 Discussion and suggestions

### 5.1 Summary of the study

As a novel form of media, social media has emerged as a primary channel through which individuals create, share and exchange opinions. Topics related to fertility, once confined to the private sphere, have gradually entered the public domain. On social media platforms, discussions are not limited to pregnancy and childbirth, they also encompass a pervasive sense of fear and anxiety surrounding fertility, leading to the widespread dissemination of fertility fear emotion.

Hypothesis 1, which posits that fertility fear emotion on social media will impact childbearing intentions, has been validated. Drawing on a comprehensive review of literature on this subject, we observe that college students are highly curious about childbirth. However, lacking formal education on fertility, they often turn to social media for information. Notably, the media frequently portrays childbirth as fraught with risks and pain. When college students are repeatedly exposed to such negative portrayals, it can easily induce anxiety and fear, resulting in vicarious trauma that subtly diminishes their desire to have children. This finding aligns with the research results of Zhu and Lei (2016), who found that the exaggerated depiction of fertility-related phenomenon on social media heightens individuals’ perception of fertility risk and reduces their willingness to have children. According to the research of Xu Jin and Zhi Mingyue, an increase of one unit in the frequency of internet use is associated with a 2.4495% decrease in individuals’ willingness to have children, and a decrease by 1.0544% decrease in their fertility intentions (Xu and Zhi, 2025). The portrayal of reproductive fear on social media facilitates the spread of fertility fear emotion between the content sharer and the viewer. This not only amplifies college students’ unease regarding their own fertility, but also intensifies their worries about the reproductive process, leading to a state of “mental sterilization”.

Hypothesis 2 posits that childbearing attitude serves as a mediating factor between fertility fear emotion and fertility intentions, a relationship that has been empirically validated. The pervasive fertility fear emotion among college students stems from a deep-seated concern about the potential losses and damages associated with having children. According to survey findings, these students perceive the current social security system as robust, allowing them to enjoy a comfortable life in their later years without the need to continue the family line. In their view, fertility is characterized as a burden that entails “negative economic effect” and “no emotional effect”.

In this context, fertility is no longer regarded as an inevitable experience or mandatory obligation. Consequently, most individuals adopt a negative attitude towards fertility. Such a negative attitude towards fertility inevitably exerts a significant influence on fertility intentions. However, it is important to note that a positive attitude towards fertility may not necessarily generate positive fertility intentions.

Hypothesis 3 asserts that subjective norms mediate the relationship between fertility fear emotion and fertility intention, and this hypothesis has been confirmed. Regarding subjective norms, Van Gelderen et al. (2008) argue that college students are at a critical stage of life development, during which the opinions of influential individuals such as internet information, parents, friends and other important people can significantly impact their attitude and decisions. College students exist within the context of family and society, and their views on fertility are shaped by the social relations they navigate. They form their own value judgments based on group norms and the expectations of those around them. When fertility fear emotion is amplified through repeated exposure and resonance on social media, college students may unconsciously succumb to the “mainstream” fertility opinions within their communities. This process can lead them to adopt the group’s negative stance on fertility, thereby intensifying their own rejection of the idea. In everyday life, college students often make choices based on the practices of those around them or the opinions of significant others. For example, research by Jin et al. (2018) confirmed that the emotional pressure stemming from parents’ fertility preferences is a powerful factor influencing women’s decisions regarding two-child family planning. From this perspective, it is evident that the fertility intentions of college students are significantly influenced by the social evaluations and expectations of those who matter most to them.

Hypothesis 4 suggests that perceived behavioral control mediates the relationship between fertility fear emotion and fertility intentions, which has been confirmed. In terms of perceived behavior control, the “fear of breeding” significantly influences college students’ self-assessment of their fertility capabilities and their confidence in their ability to successfully engage in fertility. From a research perspective, college students believe that if they have sufficient economic resources, a strong marital relationship, and a partner who is willing to share parenting responsibilities, they would be more likely to consider having children. In other words, when college students possess a higher level of confidence or a more positive assessment of their ability to engage in reproductive behavior, their desire to have children is correspondingly stronger. This indicates that college students possess a high degree of self-judgment regarding the dissemination of fertility-related information on social media. They approach the topic of future fertility in a comprehensive and concrete manner, considering various factors such as economic stability and marital harmony. If they believe they will be able to meet the conditions necessary for marriage and fertility in the future, their willingness to have children.

The Theory of Planned Behavior (TPB) has been empirically confirmed to play a mediating role in the relationship between fertility fear emotion and fertility intentions. This study demonstrates that the fertility fear emotion is significantly correlated with fertility attitude, subjective norms, perceived behavior control and fertility intention. These findings validate the applicability of the TPB to research on college students’ fertility issues in the context of social media, highlighting that fertility attitude, subjective norms and perceived behavior control are crucial factors affecting college students’ fertility intention.

The mediation pathway analysis reveals that the indirect effect of “fear of parenthood” on fertility intention through subjective norms is significantly stronger than that through fertility attitude and perceived behavioral control. In other words, the mediating effect of subjective norms is greater than that of the perceived behavioral control, which in turn is greater than that of fertility attitude. This finding aligns with the research by Zhang and Zhao (2024), but contrasts with the findings of Podsakoff et al. (2003). The discrepancy may be attributed to the fact that the “fertility fear emotion” intensified the sensitivity of college students to social expectations. Even if their personal attitude or sense of control remained unchanged, they were more inclined to follow social norms, thereby amplifying the mediating role of subjective norms compared to other factors.

Furthermore, this study identifies a chain mediation relationship among the three factors of TPB: perceived behavior control influences fertility attitude through subjective norms. Under the influence of fertility-related information on social media, college students conduct a comprehensive evaluation of their own abilities and available resources by analyzing both internal and external factors. The perception, in turn, shapes their fertility attitude. Specifically, if college students possess strong perceived behavior control, high self-efficacy, extensive knowledge reserves, and the ability to cope with external pressures, they are more likely to adopt a positive attitude towards reproduction.

### 5.2 Strategies for leveraging social media to enhance fertility rates

Based on the research findings, the author suggests that to mitigate the impact of social media driven fertility fear emotion among college students and to boost the fertility rates, more comprehensive and multi-faceted guidance is needed.

#### 5.2.1 Media perspective

Mainstream media should fully exert its leading role to construct a positive narrative space for fertility. Authoritative media ought to demonstrate discourse leadership, disseminate scientific knowledge on fertility and childcare, and popularize fertility culture as well as gender health knowledge. Mainstream media should report more successful fertility cases and produce more positive content, so as to mitigate cognitive biases arising from fragmented information dissemination via scientific information. It is necessary to gather forces of various communication stakeholders including medical experts to spread scientific knowledge of childbearing and child-rearing, break the structural imbalance of fertility-related discourse, and guide college students to establish positive and healthy fertility attitudes.

Regulatory oversight shall be imposed on the algorithm recommendation mechanisms of social media platforms, to prevent college students from being trapped in the echo chamber of fertility fear emotion caused by excessive push of homogeneous content. This will disrupt college students’ established cognitive frameworks about fertility and alleviate the fertility fear emotion triggered by information on reproductive risks.

#### 5.2.2 Government perspective

A systematic fertility support policy framework should be formulated to effectively relieve real-world fertility anxiety. In 2024, the State Council issued Several Policies on Accelerating the Improvement of the Fertility Support Policy System and Promoting the Construction of a Birth-Friendly Society; in 2025, the Implementation Plan for the Child-Rearing Subsidy System and other policy documents were released. In regulating and guiding sound fertility attitudes, the government has transformed its role from a supervisor in the past to a service provider and supporter, reversing fertility fear mentality through tangible policies.

Media platforms should maximize the potential of fertility policies, strengthen publicity on the implementation of existing fertility policies, enhance college students’ recognition of fertility policies and their identification with fertility-related remarks on social media, and foster emotional resonance between college students’ fertility attitudes and national fertility policies.

#### 5.2.3 Social perspective

A birth-friendly society should be built to systematically reduce Fertility Fear Emotion. A birth-friendly society is not only a necessity for the practical development of Chinese modernization, but also carries profound aspirations for the meaning of individual life. From a societal-wide dimension, systematic provision of institutions, construction of multi-level childcare support networks, reconstruction of the humanistic value of childbearing, and creation of an inclusive fertility culture can effectively mitigate Fertility Fear Emotion.

For families, it is essential to improve family emotional atmosphere, strengthen the positive intergenerational effect on college students’ fertility intentions, shape positive fertility attitudes through intergenerational emotional support, resolve implicit fertility resistance via equal dialogue and communication, and ease crisis narratives of fertility on social media with family well-being.

For peer groups, intergroup consensus on fertility should be reached to stimulate in-depth recognition of the beauty of life and exert the positive learning effect on fertility intentions. College students are advised to pay more attention to positive fertility and parenting concepts shared by young married individuals, so as to reduce the marginal influence of Fertility Fear Emotion spread on social media.

#### 5.2.4 Individual perspective

Individual digital literacy should be elevated to enable rational examination of fertility-related information on social media. College students ought to maintain high cognitive capacity and independent critical thinking when browsing social media. They should break information barriers through multi-faceted and multi-dimensional understanding, refrain from hasty judgment or blind identification, avoid credulous acceptance of information about reproductive risks, and refuse to be swept along by Fertility Fear Emotion.

When acquiring fertility information, individuals can obtain scientific reproductive knowledge through self-inquiry; meanwhile, they should develop the habit of in-depth reading and critical reflection, and make subjective decisions on fertility intentions only after grasping the full picture of fertility-related issues. College students should also actively participate in the production and interaction of fertility knowledge, popularize scientific fertility content via positive discourse, curb the negative dissemination of fertility-related misinformation, and jointly build a birth-friendly public opinion environment on social media.

### 5.3 Research deficiencies and prospects

While the hypotheses of this study are primarily grounded in the Theory of Planned Behavior and social media emotional tendencies, and have been empirically validated, the. The measurement of college students’ fertility intentions can also incorporate a broader range of multi-dimensional influencing factors. These factors might include economic conditions, family dynamics and cultural influences, among others. Future research could enrich the measurement dimensions of relevant variables by considering these additional aspects. However, due to the limitations in research conditions, the survey samples in this study were obtained through a sampling method, which may lack representativeness and thus limit the external validity of the findings. To address this limitation, future studies could expand the sample pool to include college students from other provinces. This would enhance the external applicability and generalizability of the research conclusions, providing a more comprehensive understanding of the factors influencing college students’ fertility intentions.

## Data Availability

The original contributions presented in the study are included in the article/supplementary material, further inquiries can be directed to the corresponding author.
